# Informatics driven quality improvement in the modern histology lab

**DOI:** 10.1093/jamiaopen/ooaa066

**Published:** 2020-11-30

**Authors:** Robert P Seifert, Vektra Casler, Nada Al Qaysi, Shaileshbhai Revabhai Gothi, Leah Williams, Patricia R Christensen, Sherri Flax, Srikar Chamala

**Affiliations:** 1 Department of Pathology, Immunology and Laboratory Medicine, University of Florida, Gainesville, Florida, USA; 2 Department of Computer and Information Science and Engineering, University of Florida, Gainesville, Florida, USA; 3 UF Health Medical Laboratories, Gainesville, Florida, USA

**Keywords:** histology, medical informatics, data visualization, surgical pathology, clinical laboratory information systems

## Abstract

Laboratory Information Systems (LIS) and data visualization techniques have untapped potential in anatomic pathology laboratories. Pre-built functionalities of LIS do not address all the needs of a modern histology laboratory. For instance, “Go live” is not the end of LIS customization, but just the beginning. After closely evaluating various histology lab workflows, we implemented several custom data analytics dashboards and additional LIS functionalities to monitor and address weaknesses. Herein, we present our experience in LIS and data-tracking solutions that improved trainee education, slide logistics, staffing/instrumentation lobbying, and task tracking. The latter was addressed through the creation of a novel “status board” akin to those seen in inpatient wards. These use-cases can benefit other histology laboratories.

**Lay summary**

Histopathology is the branch of medicine that involves the gross and microscopic examination of sampled tissue to help reach a diagnosis. Histology lab workflows incorporate a multitude of automated and manual steps that require close monitoring. Data visualization is a powerful tool that can illustrate workflow trends using a graphic representation of data. This manuscript describes data visualization and data analytic techniques leveraged to address frequent pitfalls in histology laboratory workflows. This led to multiple improvements in patient safety and quality of care. Our use-case solutions, presented here, can be adapted by other histology laboratories to overcome common laboratory challenges.

## INTRODUCTION

The microscopic examination of hematoxylin and eosin (H&E) stained slides has been the cornerstone of anatomic pathology diagnosis for over a century. Virtually every surgical specimen is processed, embedded in paraffin, cut, mounted on a slide, and stained in a histology laboratory. Anatomic pathology laboratory processes contain a multitude of technical and manual steps which can be further complicated by being located offsite. Automated instruments, particularly tissue processors and stainers, have accelerated histology lab work. However, important manual tasks remain specimen grossing, tissue cassetting, embedding, cutting, mounting, and tissue examination under a microscope for quality control. If these processes are not closely monitored, the quality of care suffers.

Most processes in a modern histology lab are captured by an integrated laboratory information system (LIS). Data visualization is a powerful tool which can illustrate workflow trends using charts, diagrams, and tables.^1–3^ The use of data visualization on captured histology data elements from the LIS grants the ability to monitor nearly all steps of histology lab operation. Careful, upstream capture of workflow steps and their corresponding data elements in the LIS can help build robust data visualizations, leading to rapid identification of process and their improvement.^3^ This improves the accuracy and timely reporting of the final pathology diagnosis, which directly affects patient care and compensation.

Our institution serves a large, roughly 1000 bed tertiary care hospital with multiple surgical subspecialties available. Diagnoses range from mundane to extraordinarily rare. Our histology laboratory also acts as an outreach lab for a number of regional clinics and is geographically separated from our main hospital and anatomic pathology offices. Our pathology department uses Beaker (Epic Systems Corporation, Verona, WI, USA), as our LIS. Beaker, as in other LIS platforms, makes use of 1D and 2D bar code reading for patient and specimen identification. Labs can use these to track specimen movement in exquisite detail and can be leveraged to evaluate anatomic pathology lab performance. We found such tracking to be essential given our lab’s geographical separation from the hospital and pathologists. To our knowledge, few authors have explored data visualization and anatomic pathology workflows. Standout work from the University of Iowa^4^ and Duke University^5^ provided our institution with guidance during our Beaker implementation. However, those authors did not fully explore the potential of data visualization for quality improvement. Using a case-oriented format, this report demonstrates data visualization techniques, as applied to anatomic pathology workflows and may serve as a guide for others.

## ENCOUNTERED CHALLENGES AND TAILORED INFORMATICS SOLUTIONS

### Histotechnologist status board

**Figure 1.**
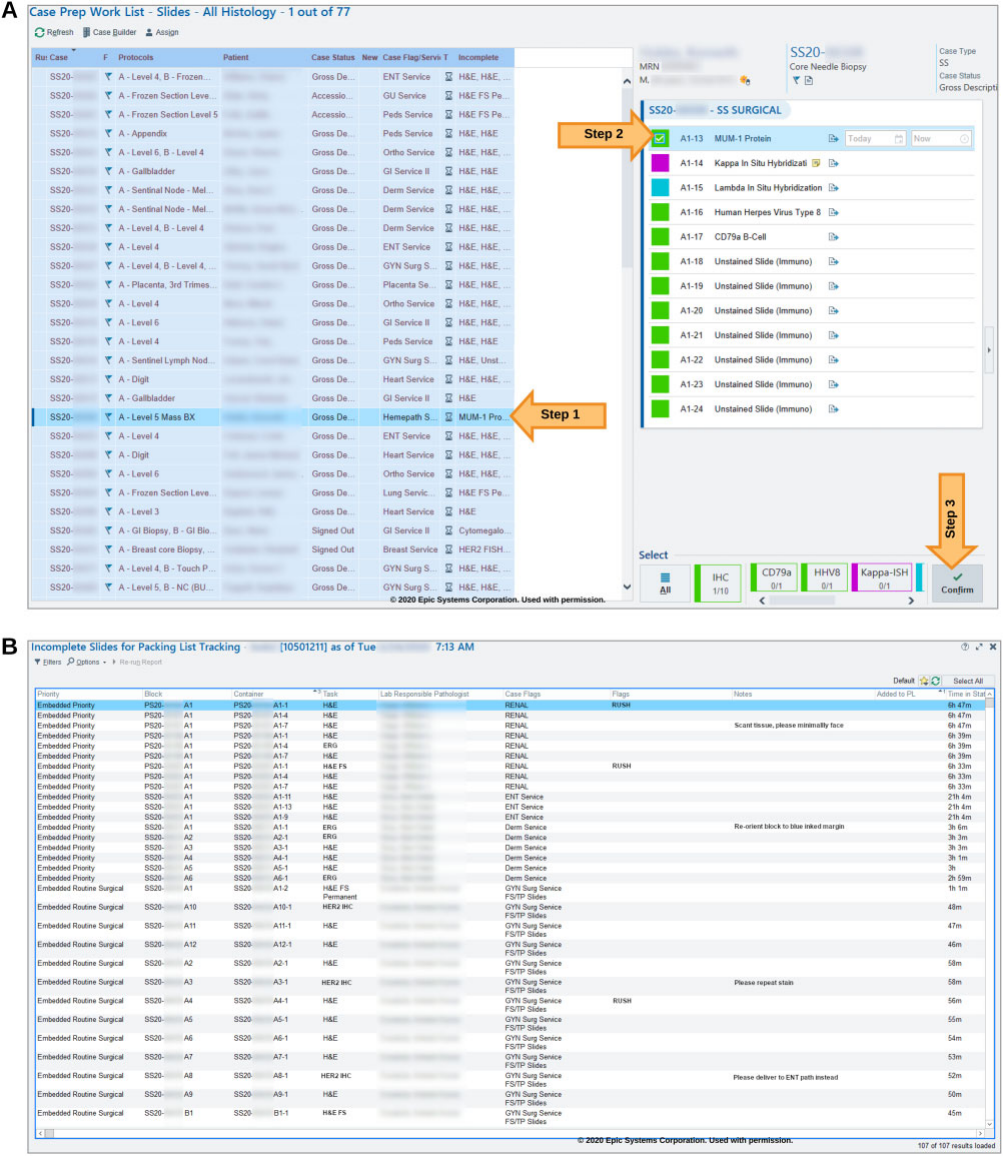
(A) The case-prep work list organizes pending histology tasks by case with a truncated list, “Incomplete” column, to show the actual pending task types. Specific tasks are “confirmed” on the right-hand panel. (B) Our custom “status board” is displayed in the lab similar to inpatient ward status boards. Here histology tasks are listed with their pertinent information, at-a-glance.

In Beaker, pending histology work is organized in a module called the “case-prep worklist.” A major deficiency in this module is that it is case-based and not task-based. Every line in the case-prep worklist represents one case, which can have multiple tasks (eg, see **Figure 1**A—H&E, MUM-1, Her2). In a complex lab, histotechnologists work on dedicated sets of tasks and do not work in a case-based manner. For example, a histotechnologist may only work on biopsy H&E slides for their shift.

Within Beaker, pending tasks are presented as a single, concatenated and truncated string within a column (see **Figure 1**A—“Incomplete” column) with no option for sorting, filtering, or expanding. Histotechnologists must click on a case in the list to view all pending tasks in a separate individual pane (see right side pane in **Figure 1**A, Steps 1 and 2). To begin work on a slide, a histotechnologist needs to mark the individual task as “confirmed” (**Figure 1**A, Step 3). At this point, a unique label is printed for the slide and the task disappears from the case-prep worklist (**Figure 1**A). This is disadvantageous since the system considers the task to be complete at this point when, in fact, the work (technical process of slide preparation) is just beginning. Also, there is minimal flexibility in changing task parameters after it is confirmed. Specific task details, such as rush priority, as well as the tasks themselves, can be “buried” amid other information. We found the overall histotechnologist user experience for the case-prep worklist unintuitive during initial Beaker implementation and felt the need for a task-centric worklist.

To address these drawbacks, we explored other workflow organization techniques implemented in EPIC modules in other areas of the hospital, namely inpatient/emergency department “status boards.” Status boards, commonly found in nursing areas, display key information regarding patient care in an organized way tailored to that specific area. With this template in mind, we generated a task-centric “status board” within Beaker for use in the histology laboratory. We implemented the status board using the Beaker MyReports Module by leveraging tailored report settings (**Figure 1**B). This status board displayed pending histology lab tasks, priorities and time-in-status among other task-specific notes that the ordering user may provide. This allowed for triage of urgent cases and helped communicate case-specific needs at-a-glance. The status board has a filter and sort features. This bolsters the user experience, visualizing all specimens that require the same task. For example, the user can filter the status board by “H&E biopsy” tasks and then sort by date received to ensure the oldest tasks are addressed first. While doing this, the user would also be able to note tasks flagged as “RUSH” as well as specific notes from the grossing staff such as “tiny biopsy, three total pieces.” Lastly, the status board could adapt to changes in slide routing, such as a case being assigned to a different pathologist while in process, which was not possible under the case-prep worklist. When slide tasks are shipped out of the lab, they are automatically removed from the status board, mimicking the actual workflow. Our status board in many ways supplanted the default case-prep worklist used by Beaker.

Our status board is also useful for intraoperative slide logistics. At our institution, all intraoperative slides accompany the tissue cassettes to histology, after specimen grossing. Ideally, all diagnostic case materials would then remain together through histology lab generation of permanent tissue sections from the cassettes. Unfortunately, intraoperative slides would be missed when staff were packing permanent sections for a given case and were not sent to the main hospital. We found that this was because there was no indicator to alert the staff to locate intraoperative slides at the time of case packing. An additional benefit of the status board is that it also acts as an inventory for all case materials which need to be shipped to the pathologist at a specific time. Thus, unlike any default Beaker module, we created a status board to display the intraoperative slides to be shipped along with the permanent sections.

### Slide volume, compensation, and resident training

**Figure 2.**
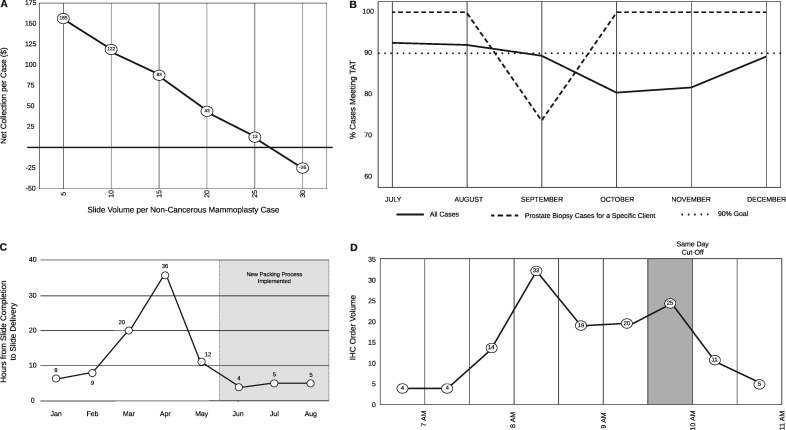
This composite figure shows a rendering of data generated by our SAP Business Objects dashboards. These graphs are continuously reviewed but, as described in their relevant sections, SAP Business Objects functionality allowed for drill-down and filtering data elements to investigate and solve various histology lab problems. (A) A plot of slides per non-cancerous mammoplasty case by compensation illustrates the inverse relationship between the number of blocks submitted and the net revenue. (B) Prostate biopsy specimens from a specific client (dashed line) and overall (solid line) histology TAT. (C) Plot of hours from slide completion in histology to slide delivery to pathologist by month. This delay was ameliorated by changing courier procedure. (D) A daily surge in IHC order volume was identified immediately prior to the 10am “same day” cutoff. This surge, and its effects on case TAT, assisted in lobbying for additional instrumentation.

Gross tissue specimens are examined by both trained professionals (pathology assistants) as well as pathologists in training (pathology residents). Certain trainee mistakes may be overlooked by mentors and staff. Routine lab volume reporting failed to identify an increase in block volumes from cases prepared by grossing-room trainees. Specifically, excess blocks were submitted for routine mammoplasty (non-cancerous) specimens by trainees. This spike in volume delayed the processing of these specimens and impeded other lab tasks on the days in which excess blocks were submitted. To evaluate the scope and severity of the problem we developed a data dashboard. This helped to identify the trainees driving the issue. Additionally, these data were used in conjunction with compensation data to visualize the drop in case compensation with the submission of excess blocks (and by extension, slides) on these types of cases (**Figure 2**A). This visualization proved useful in trainee education and corrected the blocks-per-case levels.

### Subspecialty specific turn-around time investigation

Turn-around time is calculated from the time of specimen accessioning to the time when the final pathology report is finalized. In 2019, the histology leadership was notified by Genitourinary sub-specialist pathologists that our prostate biopsy TAT was slow. This was initially misattributed to a decrease in staffing which affected the overall histology lab TAT for September to November 2019 (see **Figure 2**B). The prostate subspecialty specific drop-in TAT compliance was investigated by building a dynamic TAT reporting dashboard with the capability to filter across multiple variables (eg, surgical specialty, specimen source, contributing clinic). Using this dashboard, we filtered the cases by prostate subspecialty and client, which revealed that the delay was not due to decreased staffing but rather an idiosyncrasy in case handling. The delayed prostate specimens exclusively belonged to a specific outpatient surgical center. The case handling error was traced to the placement of the biopsies in question in a nonstandard, overlooked location. Correction of the error led to 100% TAT compliance by the following month (**Figure 2**B—October 2019).

### Delayed slide delivery

Several end-user pathologists reported that the histology lab was taking longer to complete outreach cases than in-house cases. Histology lab TAT reports showed no delays during the time period investigated. However, transit times for cases were not included in the histology TAT reports. To identify the source of delay, we extracted previously unevaluated timestamps from the EPIC Clarity database. Timestamps are recorded in this database every time a slide is scanned but they were not necessarily being used to evaluate lab efficiency.

SAP BusinessObjects was used to visually interrogate the time-delta of every step a histology slide experienced from specimen processing to individual slide delivery (eg, as slide creation, packing out or received times).

This data visualization solution quickly identified the source of delay. The delay was due to slides being placed in a sorting/packing area (in the histology lab) that was not checked by courier staff after 4pm. Thus all slides produced after 4pm missed the evening courier to surgical pathology despite being packed out of the histology lab in a timely manner. This step was not captured by routine histology TAT because the TAT captures only until a slide is scanned onto a packing list. Once identified, the error was quickly corrected and the average time to slide delivery normalized (see **Figure 2**C).

### Same-day immunohistochemistry order volume surge detection

Our lab offers same-day delivery for immunohistochemistry (IHC) orders placed before 10am. Histotechnologists felt that most IHC orders arrived just before the 10am deadline, limiting capacity by virtue of the number of instruments and insufficient staffing. We used Epic Beaker and SAP BusinessObjects to generate histograms of IHC order volume by time (**Figure 2**D). We were able to identify a surge in IHC ordering between 9:30am and 10am, corresponding to the strain experienced by histology staff. **Figure 2**D shows an example of a spike in order placement between 9:30am and 10am. Identification of order surges like this and presentation of such information in an easily digestible format to administrators, allowed us to successfully campaign for additional IHC instruments and reorganization of human resources. Being able to generate these kinds of visualizations can help lab leadership lobby for resources from hospital administration.

### Specimen packing and paperwork deviation

All the slides generated in the histology lab have a unique barcode which allows precise tracking within LIS. Slides may be sent to pathologists in other buildings in our healthcare system. Beaker LIS requires the creation of a “packing list,” which is a printable inventory of all the material (slides/blocks) being shipped from one location to another. A hardcopy of the printed packing list accompanies the specimens in transit. Items in the packing list are scanned at the destination as “received” to complete the digital paper trail. Items can only be received if the packing list (at the shipping location) was marked as “closed” prior to shipping.

Difficulties arose in our lab due to “un-closed” packing lists. For example, specimens sent from histology lab to the surgical pathology sign out area could not be scanned as received if the packing list status is not “closed” at the point of origin. This created a work stoppage at the destination, as Beaker LIS prevents “closing” a packing list if the user logged in at the destination. Correction of this error at the destination involves a convoluted process of switching contexts (i.e., virtual location) within Epic Beaker, effectively tricking the LIS into thinking one is at the point of origin (histology lab) instead of the destination (surgical pathology). After marking a packing list as “closed,” the user must switch the context again to receive the slides. This created needless delays in patient care and frustration on the part of the technologists owing to the frequency of this error.

Our solution involved the addition of a large warning to the printed hard copy of a packing list. Now when a packing list is printed while “un-closed,” a large warning appears at the top declaring “DRAFT DO NOT USE” to the specimen origin technologists. This simple addition serves as a useful reminder to “close” the packing list prior to the specimen leaving the location and has greatly reduced the incidence of this error.

## CONCLUSION

LIS solutions are an integral part of any modern anatomic pathology lab. However, “canned” LIS implementation modules cannot fit every laboratory’s needs. Our solutions demonstrated creative use of common LIS tools (Table 1). The technical and/or functionality framework that we demonstrated in this manuscript could be adapted by other institutions to address common problems encountered by anatomic pathology laboratories. We feel our implementations, while specific to our institution’s needs, are creative and nuanced in ways that may benefit other groups. Careful and continuous evaluation, post go-live, along with creative problem solving are required to fully realize the potential of electronic data tracking, ultimately leading to meaningful insights. This includes improving workflows and data analytics. Inclusive collaboration across all stakeholders, which comprises not only laboratory leadership but also “ground level” staff, is required to bridge workflow knowledge gaps with data analytics. Additionally, our laboratory’s stakeholders became more conversant in informatics approaches through a specialized departmental pathology informatics courses.^6^ Our partnership led to the identification and resolution of multiple workflow issues that could arise in any modern histology lab. It is our hope that other histology lab leaders can find utility and inspiration from our experience.

**Table 1.** Lessons learned

Histotechnologist status board	Nonstandard reporting functions can be customized to display information in an efficient, workflow-oriented manner. This status board is used continuously by staff.
Slide volume, compensation and resident tracking	Resident training and policy can be developed through the utilization of histology block and slide volume data. Conclusions from these data are now part of resident training.
Subspecialty specific TAT	Careful slicing of data with visualization can unveil TAT problems. Specialty specific reports are utilized as a part of monthly QC and are part of any TAT related investigation in the lab.
Delayed slide delivery	Not all steps between initial lab processing of tissue and delivery to pathologist are captured by routine TAT reports. Lab to Pathologist TAT visualizations are part of any TAT related investigation in the lab.
Same day IHC volume surge detection	Surges in volume related to a specific lab area can be visualized with good informatics practices. This report is used during meetings with administration regarding acquisition of new instruments or personnel as well as staff scheduling.
Specimen packing and paperwork deviation	Creative use of LIS features can provide an extra layer of patient safety. These alerts have reduced the incidence of the type of error described to near zero.
